# Efficacy and safety of sorafenib in elderly patients with advanced hepatocellular carcinoma

**DOI:** 10.6061/clinics/2021/e2498

**Published:** 2021-01-18

**Authors:** Guilherme Nader Marta, Leonardo G. da Fonseca, Maria Ignez Braghiroli, Fernando Moura, Paulo M. Hoff, Jorge Sabbaga

**Affiliations:** Instituto do Cancer do Estado de Sao Paulo (ICESP), Hospital das Clinicas (HCFMUSP), Faculdade de Medicina, Universidade de Sao Paulo, Sao Paulo, SP, BR

**Keywords:** Hepatocellular Carcinoma, Older Adults, Sorafenib, Safety, Efficacy

## Abstract

**OBJECTIVES:**

To evaluate the efficacy and safety of sorafenib in elderly patients with advanced hepatocellular carcinoma (HCC).

**METHODS:**

We analyzed data from a cohort of patients with advanced HCC treated using systemic treatment according to the local institutional protocol. Patients were divided into two groups, Group A, individuals <70 years of age, and Group B, individuals 70 years of age or older at the time of treatment initiation. Efficacy, measured based on overall survival (OS) and time to treatment failure (TTF), and toxicity were compared between groups.

**RESULTS:**

A total of 238 patients with advanced HCC who received sorafenib between 2007 and 2018 were evaluated. The median age for Group A was 59.1 years and that for Group B 73.6 years. The major prognostic characteristics were balanced between the groups. There were no significant differences in OS between Group A (8.0 months, 95%CI 6.34-9.3) and Group B (9.0 months, 95%CI 5.38-12.62), *p*=0.433, or in TTF between Group A (3.0 months, 95%CI 2.39-3.60) and Group B (3.0 months, 95%CI 1.68-4.32), *p=*0.936. There were no significant differences between Groups A and B with respect to the incidence of adverse events or treatment discontinuation because of toxicity.

**CONCLUSION:**

Efficacy and safety of sorafenib did not differ significantly between younger and older patients with HCC. Our data suggest that age alone should not restrict clinical decision-making for patients with advanced HCC.

## INTRODUCTION

Approximately 70% of the patients with cancer are aged 65 years and older, and the number of patients with cancer in this age group is projected to significantly increase over the next decades (1). Hepatocellular carcinoma (HCC) is the fourth leading cause of cancer-related deaths worldwide (2). Although the mean age of HCC presentation is between 50 and 60 years (3), a substantial proportion of HCC patients is older. Sorafenib was the first systemic agent to improve survival and has been the backbone of advanced HCC treatment over the past decade (4). Recently, an expansion in systemic treatment options (5) has increased the complexity of therapeutic decisions for the management of HCC. This scenario is even more complex in older patients because of the scarce representation of this population in clinical trials. Furthermore, concomitant advanced age with chronic liver disease increase concerns about toxicity and clinical benefits in this subgroup (6). Therefore, it is of utmost importance to broaden the available evidence to support the management of elderly patients with advanced HCC. Here, we analyzed a cohort of HCC patients with the aim of evaluating the efficacy and safety of sorafenib in this specific population.

## MATERIAL AND METHODS

### Study Design and Participants

We retrospectively evaluated a cohort of advanced HCC patients treated between October 2007 and January 2017 at the Instituto do Câncer do Estado de São Paulo (ICESP), Universidade de São Paulo, Brazil. All patients included in the analysis met the diagnostic criteria for HCC based on radiological and/or histological findings (7). Clinical characteristics related to the underlying liver disease, information on treatments, and clinical outcomes were collected from medical records at the beginning of the systemic therapy.

Patients were excluded in case of: (1) diagnosis of fibrolamellar carcinoma, (2) exposure to systemic therapy for less than 30 days, (3) insufficient data in medical records, or (4) loss to follow-up, long enough to impair outcome assessment.

Patients were divided into two groups, (Group A) individuals <70 years of age and (Group B) individuals who were 70-years old or older at the time of initiation of sorafenib treatment. Efficacy and safety were compared between Groups A and B. The study was approved by the institutional ethics committee (3.807.496).

### Treatment

According to the institutional protocol, patients who are candidates for systemic treatment receive first-line therapy with sorafenib at a dose of 400 mg twice daily, and this is continued until evidence of disease progression, unacceptable adverse events, or death. The use of reduced doses was permitted both from the beginning of the treatment and during the course of treatment (depending on tolerability and side effects) at the discretion of the treating physician. The follow-up consisted of regular clinical visits and laboratory assessments every three to four weeks and assessment of radiological response (computed tomography or magnetic resonance) every eight to 12 weeks. After sorafenib discontinuation, patients received best supportive care or were enrolled in clinical trials whenever available in the institution.

### Statistical Analysis

Overall survival (OS) was calculated from the first day of treatment until death from any cause. Patients without the event were censored at the time of the last follow-up. Time to treatment failure (TTF) because of disease progression or toxicity was calculated from the first to the last day on sorafenib.

Categorical variables were compared using the χ^2^-test or Fischer’s exact test, where appropriate. TTF and OS were estimated using the Kaplan-Meier method and curves were compared using the log-rank test. Univariate and multivariate analyses using the Cox proportional hazards model were performed to evaluate the prognostic factors. Variables were included in the multivariate analyses if they presented a p-value <0.05 in the univariate analysis and were not associated with each other. Statistical analyses were performed using SPSS software, version 22.0 (IBM SPSS Chicago, IL). A *p*-value <0.05 was considered significant. 

## RESULTS

### Patient Characteristics

From October 2007 to January 2017, 238 patients with advanced HCC who were not suitable candidates for—or had progressed after—locoregional therapies received first-line sorafenib at Instituto do Câncer do Estado de São Paulo (ICESP) and were included in the present analysis. The median age for Group A was 59.1 years and for Group B 73.6 years. The proportion of patients with preserved performance status (ECOG performance status 0) was significantly higher in Group A than that in Group B (75.3% *vs* 56.8%, *p=*0.014), respectively. The distribution of the etiologies of chronic liver diseases was homogeneous between the groups, although a higher proportion of patients with hepatitis C virus infection was observed in Group A (51.5% *vs* 27.3%, *p=*0.004). Important prognostic markers, such as staging according to the Barcelona Clinic Liver Cancer (BCLC) staging system (*p=*0.831), the presence of elevated α-fetoprotein levels (*p=*0.481), or the presence of extrahepatic spread of the disease (*p=*0.115) were balanced between groups. Locoregional treatments performed prior to systemic therapy had been delivered to 46.4% of patients in Group A and to 43.1% in Group B. Patients in Group B were more likely to receive sorafenib at reduced doses (<800 mg daily) than patients in Group A (27.3% *vs* 11.3%, *p=*0.006). The demographic and treatment characteristics are presented in Table 1.

### Efficacy and Adverse Events

**Figure 1:**
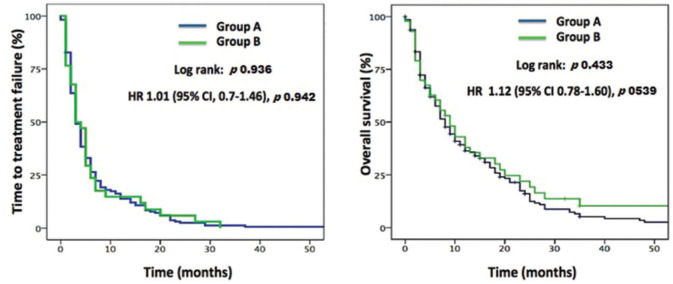
Time to treatment failure and overall survival curves for patients with advanced HCC under 70 years of age (Group A) and for patients 70 years of age or older (Group B) treated with sorafenib in the first-line setting.

At the time of the final analysis, 156 (92.9%) deaths occurred in Group A and 28 (90.3%) in Group B. The median follow-up period was 7.0 months. No significant difference was observed in TTF between Group A (3.0 months, 95%CI 2.39-3.60) and Group B (3.0 months, 95%CI 1.68-4.32), *p=*0.936. Similarly, no significant difference was observed in the median OS between Group A (8.0 months, 95%CI 6.34-9.3) and Group B (9.0 months, 95% CI 5.38-12.62), *p=*0.433 (Figure 1).

The incidence of dermatological adverse events (38.5% *vs* 33.3%, *p=*0.532), hypertension (6.9% *vs* 4.9%, *p=*0.746), and diarrhea (37.8% *vs* 26.8%, *p=*0.186) did not differ significantly between Groups A and B, respectively. Intolerance leading to sorafenib discontinuation occurred in 11.3% and 9.1% of the patients in Groups A and B, respectively (*p=*0.794). Temporary interruptions of at least 1 week were observed at least once in 6.7% of the patients in Group A *versus* 13.6% of the patients in Group B (*p=*0.131). The distribution of the most common toxicities is described in Table 2.

### Factors associated with OS in patients with advanced hepatocellular carcinoma

The variables included in the univariate analysis were age, Eastern Cooperative Oncology Group performance status (ECOG PS) etiology of hepatopathy (HCV, HBV, alcohol), initial dose of sorafenib, BCLC stage, Child-Pugh score, presence of extrahepatic metastasis, and serum levels of alpha-fetoprotein. In the univariate analysis, ECOG PS (HR 1.45, 95% CI 1.1-2.0, *p=*0.021), Child-Pugh (HR 2.93, 95% CI 1.9-4.5, *p*<0.001), and AFP levels (HR 1.53, 95% CI 1.1-2.0, *p=*0.004) achieved the significance threshold for inclusion in the multivariate analysis.

In the multivariate analysis, ECOG PS (HR 1.42, 95% CI 1.1-1.9, *p=*0.025), Child-Pugh (HR 2.59, 95% CI 1.7-3.9, *p*<0.001), and AFP levels (HR 1.48, 95% CI 1.1-1.9, *p=*0.006) exhibited a significant association with poor OS. Table 3 summarizes the results of the univariate and multivariate analyses.

## DISCUSSION

Our study focused on assessing the impact of age on the efficacy and safety of sorafenib in patients with advanced HCC. Although patients over 70 years of age more frequently received sorafenib with dose reductions, no significant OS or TTF differences were observed between patients over 70 years of age and their younger counterparts. Similarly, the frequency of adverse events occurred at comparable rates between the groups. In multivariate analysis, known prognostic factors such as ECOG PS 1-2 and Child-Pugh B and elevated serum levels of AFP (>400 ng/mL) were associated with lower survival in the entire cohort.

Liver cancer was the sixth most commonly diagnosed cancer and the fourth leading cause of cancer-related deaths worldwide in 2018 (2). The incidence and mortality rates of HCC are increasing in many parts of the world, including North America, Latin America, and central Europe (8,9). Although the median age for diagnosis of HCC in the United States is 62 years, more than 30% of patients are currently diagnosed at >70 years of age (10), emphasizing the need for data that support the clinical management of this population.

Upon using a cutoff point of 70 years of age, we did not find a significant difference in the clinical outcomes, i.e., OS and TTF between younger and older patients who received sorafenib for advanced HCC. The absence of difference between age groups becomes even more pronounced when we consider that the group of younger patients (Group A) more frequently received full doses of sorafenib (800 mg daily) and was enriched with a higher proportion of patients with preserved performance status, a known favorable prognostic factor (11), in addition to presenting a higher proportion of patients with HCC because of hepatitis C infection, a subgroup that was shown to derive greater benefit from sorafenib treatment (12).

Although different age cutoff points have been used, other authors have already sought to evaluate the efficacy of sorafenib in older patients with advanced HCC. Novel treatment strategies such as the combination of atezolizumab plus bevacizumab, have been shown to be associated with a favorable safety profile and a meaningful benefit in adults ≥65 years of age in a subgroup analysis in the IMbrave150 trial (13).

Table 4 summarizes the findings of studies comparing the clinical outcomes of young and old patients with HCC who received first-line sorafenib. Heterogeneity in survival across different cohorts is likely to reflect variations in epidemiological and clinical contexts, including improved management of treatment-related adverse events with tailored dosing (14). Most of these studies also did not find any significant correlation between age and OS subgroups (15-21). Hajiev et al. analyzed a large cohort of 5598 patients, including 792 patients aged ≥75 years and found equivalent outcomes in the groups < or ≥75 years, independent of dose (15). In this cohort, a higher rate of discontinuation because of toxicity in the older subgroup was observed, which indicates that physicians may be inclined to discontinue treatment in older patients who experience an adverse event, fearing further severe complications. In addition, they observed that patients aged ≥75 years who received sorafenib had lower alpha-fetoprotein levels, less portal invasion, and better liver function. This probably reflects the reason why physicians tend to prescribe sorafenib for older patients because of these favorable prognostic factors (15).

In contrast, Morimoto et al. (18) reported a survival benefit in favor of the subgroup of younger patients. In this respect, the following limitations of this study should be taken into consideration when interpreting the results: (1) this study included a relatively small population (n=76) comprising exclusively of Asian patients; (2) treatment compliance was poor (less than 50% of patients received more than 80% of the prescribed dose of sorafenib); and (3) the median duration of treatment was short (only 1.7 months), possibly because of a high rate of adverse events.

In the present analysis, although older patients more frequently received lower doses of sorafenib, such dose reductions did not significantly impact efficacy or toxicity. The prescription of a reduced initial dose in 27% of our cohort might reflect a common perception of physicians that elderly patients are at increased risk of adverse events. In addition, a higher proportion of ECOG PS 2 among the older group might also explain this finding.

Aging has been associated with changes in the pharmacokinetics of antineoplastic agents because of a number of age-related changes, including modifications in renal function and excretion, changes in liver function and metabolism, drug absorption and distribution alterations, among others (22-24). For this reason, the doses of drugs provided to younger patients are not necessarily extrapolatable to older patients. In addition—specifically regarding the use of sorafenib in the treatment of HCC—recent evidence suggests that no significant efficacy impairment might be observed when starting treatment at doses below 800 mg daily (25).

Older cancer patients are often at a higher risk of “undertreatment” because of exaggerated clinical estimation of their frailty, as well as “overtreatment” because of inappropriate evaluation of their fitness to receive intensive treatment regimens (26,27). As reinforced by our data, age alone is not a reliable predictor of efficacy or adverse events in advanced HCC patients treated with sorafenib. Specific geriatric assessment tools that are not widely used in routine oncology assessments have been validated to predict adverse outcomes in patients being considered for cancer treatments (26). Evidence suggests that geriatric assessment should include—at a minimum—evaluation of function, comorbidity, falls, depression, cognition, and nutrition (28). Finally, the underrepresentation of patients over 65 years in clinical cancer trials is a major issue because it weakens the evidence supporting the therapeutic decisions in this population (29,30). Thus, the use of inclusion or exclusion criteria based exclusively on age should be discouraged, and clinical trials in oncology should be performed without an upper age limit to allow inclusion of eligible older adults (31).

It has been demonstrated that specific types of adverse events have a prognostic value, such as skin toxicities and hypertension (32). A longer follow-up of the present cohort can aid us in understanding whether these observations apply to the elderly population, as the incidence of some specific events in this study was low. Further, worse liver function is a key prognostic factor in advanced HCC (33) and is of particular relevance in older patients, as suggested by our multivariate analysis.

Our study has limitations that should be considered when interpreting the results. This was a single-center retrospective analysis, which may hinder the external validity of our results. We excluded patients with exposure to systemic therapy for less than 30 days to minimize the inclusion of patients with premature loss to follow-up, which may have underrepresented patients with rapidly progressive disease. In addition, because of its retrospective nature, there was no randomization between the treatment arms, which may have influenced our outcomes.

## CONCLUSIONS

Although older patients received reduced starting doses of sorafenib and presented lower performance status at the start of systemic treatment, these patients did not exhibit a significant difference in OS and TTF in comparison with younger patients in the present retrospective analysis. The safety of sorafenib treatment was similar between younger and older patients, with similar toxicity-related discontinuation rates between groups. Our results suggest that age alone should not be used to determine the therapeutic strategy for patients with advanced HCC being considered for sorafenib therapy.

## CONFLICTS OF INTEREST

Guilherme Nader Marta has received travel/accommodation grants from Bayer Schering Pharma and Roche. Leonardo Gomes da Fonseca received lecture fees from Bayer and travel grants from Bayer and Ipsen. Maria Ignez Braghiroli received grants from Merck Sharp and Dohme and Bristol-Myers Squibb for clinical trials at her institution and from Merck Sharp and Dohme, Bayer, and Astra Zeneca for being a member of their advisory boards. Paulo M. Hoff has no conflicts of interest to declare. Jorge Sabbaga received grants from Bayer for being a member of its advisory board.

## AUTHOR CONTRIBUTIONS

Marta GN, Fonseca LG, Sabbaga J contributed in study concepts. Fonseca LG, Braghiroli MI contributed in study design. Marta GN, Fonseca LG, Moura F contributed in data acquisition. Braghiroli MI, Hoff PM, Sabbaga J contributed in quality control of data and algorithms. Marta GN, Fonseca LG, Sabbaga J contributed in data analysis and interpretation. Marta GN, Fonseca LG, Moura F contributed in statistical analysis. Marta GN, Fonseca LG contributed in manuscript preparation. Moura F, Braghiroli MI, Hoff PM, Sabbaga J contributed in manuscript editing. Braghiroli MI, Hoff PM, Sabbaga J contributed in manuscript review.

## Figures and Tables

**Table 1:** Baseline patient demographics and disease characteristics at the beginning of systemic treatment.

	Total	Group A <70 yr	Group B ≥70 yr	
Number of patients (%)	N=238 (100)	N=194 (81.5)	N=44 (18.5)	*p*-value
Sex [n (%)]				
Male	189 (79.4)	154 (79.4)	35 (79.5)	
Female	49 (20.6)	40 (20.6)	9 (20.5)	
Age, years				
Mean Age (SD)	61 (10.5)	57.8 (8.9)	74.8 (3.9)	<0.001
Median Age, years (range)	61.8 (18.9-84.9)	59.1 (18.9-69.7)	73.6 (70.0-84.9)	<0.001
ECOG PS [n (%)]				
0	171 (71.8)	146 (75.3)	25 (56.8)	0.014
1	55 (23.1)	44 (22.7)	11 (25.0)	0.742
2	12 (5.0)	4 (2.1)	8 (18.2)	<0.001
Underlying CLD [n (%)]				
Hepatitis C	112 (47.1)	100 (51.5)	12 (27.3)	0.004
Hepatitis B	46 (19.3)	37 (19.1)	9 (20.5)	0.834
Alcohol-related CLD	36 (15.1)	27 (13.9)	9 (20.5)	0.275
NAFLD	19 (8.0)	13 (6.7)	6 (13.6)	0.131
Other	25 (10.5)	17 (8.8)	8 (18.1)	0.097
Extrahepatic disease [n (%)]	112 (47.1)	96 (49.5)	16 (36.4)	0.115
AFP ≥400 ng/mL [n (%)]	125 (52.5)	104 (53.6)	21 (47.7)	0.481
Child-Pugh score [n (%)]				0.664
A	205 (86.1)	168 (86.6)	37 (84.1)	
B	33 (13.9)	26 (13.4)	7 (15.9)	
Local treatments [n (%)]				
Transplant	13 (5.5)	12 (6.2)	1 (2.3)	0.472
Resection	28 (11.8)	22 (11.3)	6 (13.6)	0.686
Radiofrequency ablation	10 (4.2)	10 (5.2)	0	0.215
TACE	71 (29.8)	58 (29.9)	13 (29.5)	0.963
BCLC stage [n (%)]				
A	46 (19.3)	38 (19.6)	8 (18.2)	0.831
B	41 (17.2)	30 (15.5)	11 (25.0)	0.130
C	151 (63.4)	126 (64.9)	25 (56.8)	0.312
Sorafenib dose [n (%)]				
Initial dose 400 mg BID	204 (85.7)	172 (88.7)	32 (72.7)	0.006
Initial reduced dose	34 (14.3)	22 (11.3)	12 (27.3)	0.006

ECOG PS (Eastern Cooperative Oncology Group performance status); CLD (chronic liver disease); NAFLD (nonalcoholic fatty liver disease); APF, α-fetoprotein; TACE (transarterial chemoembolization); BCLC (Barcelona Clinic Liver Cancer); BID, twice a day.

**Table 2:** Adverse events (any grade) most commonly reported by patients and severe toxicities leading to discontinuation of sorafenib.

	Group A	Group B	*p*-value
Skin rash	72 (38.5)	14 (33.3)	0.532
Hypertension	13 (6.9)	2 (4.9)	0.746
Diarrhea	71 (37.8)	11 (26.8)	0.746
Toxicities leading to discontinuation	22 (11.3)	4 (9.1)	0.794

**Table 3:** Univariate and multivariate analyses of factors associated with overall survival.

	Unvariate		Multivariate	
Baseline variables	HR (95% CI)	*p*-value	HR (95% CI)	*p*-value
Age (<70 yr *vs* ≥70 yr)	0.81 (0.5-1.2)	0.268		
ECOG PS (0 *vs* 1-2)	1.45 (1.1-2.0)	0.021	1.42 (1.1-1.9)	0.025
Hepatitis C (no *vs* yes)	0.77 (0.5-1.1)	0.190		
Hepatitis B (no *vs* yes)	0.89 (0.6-1.4)	0.615		
Alcohol-related CLD (no *vs* yes)	1.26 (0.8-2.0)	0.332		
Sorafenib dose-daily (800 mg *vs* <800 mg)	1.28 (0.8-1.9)	0.220		
BCLC stage (A-B *vs* C)	1.22 (0.9-1.7)	0.218		
Child-Pugh (A *vs* B)	2.93 (1.9-4.5)	<0.001	2.59 (1.7-3.9)	<0.001
Extrahepatic disease (no *vs* yes)	1.14 (0.8-1.5)	0.363		
AFP (<400 *vs* ≥400 ng/mL)	1.53 (1.1-2.0)	0.004	1.48 (1.1-1.9)	0.006

ECOG PS (Eastern Cooperative Oncology Group performance status); CLD (Chronic Liver Disease); BCLC (Barcelona Clinic Liver Cancer); APF, α-fetoprotein. HR (hazard ratio); CI (confidence interval).

**Table 4:** Studies comparing the clinical outcomes of younger and older patients with HCC treated with sorafenib.

	Age cutoff	Sample size	OS, months (younger *vs* older)	*p*-value
Hajiev et al. [15]	75 years old	5598	7.3 *vs* 7.2	0.97
Wong H et al. [16]	70 years old	309	5.32 *vs* 5.16	0.310
Edeline J et al. [17]	70 years old	129	9.6 *vs* 12.6	0.250
Morimoto M et al. [18]	75 years old	76	NR^a^	0.022
Ziogas et al. [19]	75 years old	190	7.1 *vs* 10.4	0.360
Nishikawa H et al. [20]	75 years old	458	9.7 *vs* 8.2	0.641
Jo M et al. [21]	80 years old	185	10.5 *vs* 11.7	0.450

a Not reported.
